# Societies Drifting Apart? Behavioural, Genetic and Chemical Differentiation between Supercolonies in the Yellow Crazy Ant *Anoplolepis gracilipes*

**DOI:** 10.1371/journal.pone.0013581

**Published:** 2010-10-22

**Authors:** Jochen Drescher, Nico Blüthgen, Thomas Schmitt, Jana Bühler, Heike Feldhaar

**Affiliations:** 1 Department of Animal Ecology and Tropical Biology, University of Würzburg, Würzburg, Germany; 2 Department of Evolutionary Biology and Animal Ecology, Faculty of Biology, University of Freiburg, Freiburg, Germany; 3 Department of Behavioural Physiology and Sociobiology, University of Würzburg, Würzburg, Germany; 4 Department of Behavioural Biology, University of Osnabrück, Osnabrück, Germany; Field Museum of Natural History, United States of America

## Abstract

**Background:**

In populations of most social insects, gene flow is maintained through mating between reproductive individuals from different colonies in periodic nuptial flights followed by dispersal of the fertilized foundresses. Some ant species, however, form large polygynous supercolonies, in which mating takes place within the maternal nest (intranidal mating) and fertilized queens disperse within or along the boundary of the supercolony, leading to supercolony growth (colony budding). As a consequence, gene flow is largely confined within supercolonies. Over time, such supercolonies may diverge genetically and, thus, also in recognition cues (cuticular hydrocarbons, CHC's) by a combination of genetic drift and accumulation of colony-specific, neutral mutations.

**Methodology/Principal Findings:**

We tested this hypothesis for six supercolonies of the invasive ant *Anoplolepis gracilipes* in north-east Borneo. Within supercolonies, workers from different nests tolerated each other, were closely related and showed highly similar CHC profiles. Between supercolonies, aggression ranged from tolerance to mortal encounters and was negatively correlated with relatedness and CHC profile similarity. Supercolonies were genetically and chemically distinct, with mutually aggressive supercolony pairs sharing only 33.1%±17.5% (mean ± SD) of their alleles across six microsatellite loci and 73.8%±11.6% of the compounds in their CHC profile. Moreover, the proportion of alleles that differed between supercolony pairs was positively correlated to the proportion of qualitatively different CHC compounds. These qualitatively differing CHC compounds were found across various substance classes including alkanes, alkenes and mono-, di- and trimethyl-branched alkanes.

**Conclusions:**

We conclude that positive feedback between genetic, chemical and behavioural traits may further enhance supercolony differentiation through genetic drift and neutral evolution, and may drive colonies towards different evolutionary pathways, possibly including speciation.

## Introduction

In most social insect species, a colony is a closed family unit that contains a queen and her daughter workers [1]. The queen lays eggs, which are cared for and reared by the workers. As the colony reaches maturity, males and virgin queens are produced which mate with reproductives from other colonies in periodic mating events. Soon after copulation, the males die while the fertilized queens disperse in an attempt to independently found new colonies. As a consequence of this strategy, gene flow among colonies is maintained within the population.

Social insects such as ants, however, show a large variety in social organization, reproductive systems, mating behaviour and dispersal modes. Some tramp ant species such as *Linepithema humile* [2], *Pheidole megacephala* [3], *Wasmannia auropunctata* [4], *Lasius neglectus* [5] or *Anoplolepis gracilipes* [6] share a similar set of strategies: They form large polydomous, highly polygynous supercolonies, within which aggression is absent and individuals move freely between nests [7]. Supercolonies differ from other polygynous and polydomous ant colonies, in that they are usually too large to allow direct cooperative interactions of individuals from distant nests [8]. In contrast to most other ant species, nuptial flights of supercolony-forming ant species are rare or even absent, and mating often takes place within the nest or the supercolony. After fertilization, queens stay within the maternal nest, move to other nests within the supercolony or disperse at the fringe of the supercolony through budding, i.e. the occupation of a suitable nest by one or several queens that are accompanied by workers and sometimes brood from the colony the queens themselves originated from. As a consequence of these mating and dispersal strategies, gene flow between supercolonies may be extremely limited or absent, as has been pointed out by studies on *L. humile* [2], [8], [9] and *A. gracilipes* [10]. The study by Thomas *et al.* [10] strongly suggests that gene flow is absent between two sympatric and mutually aggressive *A. gracilipes* supercolonies on Christmas Island, Indian Ocean. The two supercolonies were genetically differentiated both in nuclear and mitochondrial loci. Furthermore, intranidal mating in *A. gracilipes* can be frequently observed (in laboratory colonies of *A. gracilipes*) and workers are highly aggressive towards virgin queens and males from other supercolonies (Drescher, Feldhaar, pers. obs.), suggesting that gene flow among supercolonies may be prevented by behavioural barriers. As a consequence, lack of gene flow between ant colonies should lead to genetic differentiation among them.

Nestmate recognition in ants usually relies on cuticular hydrocarbons (CHC's) [11], [12], [13], [14], [15] although other substance classes can also be involved, such as fatty acids [16]. CHC's are largely genetically determined [17], [18], [19], [20], [21], which implies that genetic differentiation among sympatric ant colonies may entail the diversification of genetically based CHC profile compounds through genetic drift and the accumulation of supercolony-specific mutations. Thus, in ant species with strict intranidal mating, CHC-profiles may differ between colonies not only in terms of relative abundances of epicuticular compounds (quantitative differences, as is the case in most ant species with intercolonial mating), but also in the composition of the profile itself (qualitative differences).

As the origin and future of supercoloniality in ants is unclear [22], studying the degree and nature of differentiation within and between supercolonies may help in understanding the evolutionary paths that those species might take [22]. We thus measured patterns of behavioural, genetic and chemical differentiation within and between six spatially separated supercolonies in north-eastern Borneo, and discuss the data with respect to potential evolutionary trajectories of *A. gracilipes*. As suggested by previous studies, NE-Borneo is inhabited by a mosaic of variably related, ecologically dominant *A. gracilipes* supercolonies [23], [24] with unusually high intracolonial relatedness estimates compared to all other supercolonial ant species studied so far [22], [24]. Thus, assuming that the scenario described above applies to *A. gracilipes*, we expect that the varying genetic differentiation between supercolonies corresponds to the degree of CHC-profile differentiation. As intranidal mating may be the dominant, if not exclusive, reproductive strategy in *A. gracilipes*, we furthermore expect that CHC-profiles differ both quantitatively as well as qualitatively between supercolonies, and that qualitative differences between the CHC-profiles of supercolonies are independent of substance classes as would be expected by an accumulation of random, supercolony-specific mutations.

## Materials and Methods

### Selection and maintenance of colonies

We localized six *Anoplolepis gracilipes* supercolonies within the study area around Poring Hot Springs, Sabah, Malaysia (6°04′ N, 116°70′ E, colonies are from here on referred to as supercolonies P1-P6, Figure S1). All supercolonies included in this study were at least 200 m in diameter, except for one slightly smaller supercolony (P1), which spanned only 100 m (Figure S1). The supercolonies were between 150 m (P1-P2) to ca. 15 km (P6-P5) apart. Workers, brood and queens from a single nest per supercolony were collected and transferred into plastic containers (45×25×25 cm) treated with Fluon™ to prevent escape (henceforth termed subcolony). All subcolonies were offered newspaper as nesting material and were fed water, honey and tuna every third day. Each of the subcolonies contained at least 1500 workers, 200 pupae/larvae and 3–12 queens (except for P1, which contained about 500 workers, 100 pupae and two queens).

### Behavioural assays

Behavioural assays were performed within and between each of the six subcolonies (15 intercolonial combinations) using two different indices (Aggression Index *AI* and Mortality Index *MI*). Both indices were measured by placing 5 individuals of each colony (10 ants per trial per pairwise colony combination) inside a Fluon™ coated plastic cylinder (diameter = 10 cm, height = 5 cm) on a sheet of paper which was replaced after each trial. The average maximum level of aggression (henceforth termed aggression index *AI*) was obtained as the average of the most aggressive interaction within 5 minutes across ten replicates according to the following categories: 1 – ants displayed no reaction towards each other/no physical contact, 2 – reciprocal antennation, 3 – ants biting and spread-eagling one another, 4 – ants protruding gaster/spraying formic acid while biting opponent. Aggression levels 1 and 2 were considered nonaggressive while aggression levels 3 and 4 were considered antagonistic/aggressive. In order to measure the Mortality Index *MI* (as described in [23]), encounters between five workers of each subcolony were observed for a period of 60 minutes. Every minute, the number of dead individuals was counted. For each trial, the mortality index *MI* was obtained as *MI* = (y/2)/t50, with y being the total number of individuals killed at the end of the experiment (60 min) and t50 the time (in steps of 1 min) when half of this number (y/2) was already killed. Thus, this index allowed us to describe aggression by combining both the number of dead individuals as well as the speed at which ants kill each other.

Additionally, we measured aggression of workers towards allocolonial sexuals, i.e. virgin alate queens and males (Text S1).

### Relatedness and population structure

Workers from each supercolony were sampled in 98.8% EtOH p.A. 20 workers from each of the six subcolonies were genotyped using six polymorphic microsatellite loci (Ano1, Ano3, Ano4, Ano6, Ano8, Ano10) according to the protocol in Feldhaar *et al.* [25]. Genetic diversity within supercolonies was measured by genotyping five workers of two to four additional nests per supercolony (two additional nests for P1 and P5, four nests for P2, P3, P4 and P6). This resulted in a total number of 220 genotyped individuals.

Relatedness within and between the six supercolonies was calculated using Relatedness 5.0.8 [26], including all individuals as reference population. All *R* values presented in this study arise from the half-matrix resulting from 220 × 220 pairwise comparisons of individual relatedness excluding comparisons of each individual with itself. Secondly, we conducted a three-level hierarchical analysis of molecular variance (AMOVA) over the entire data set to determine how genetic variability was distributed across three levels (between individuals within nests, among nests within a supercolony, among supercolonies) in the population using ARLEQUIN 3.01 [27]. Thirdly, we performed assignment tests using STRUCTURE 2.2 [28], [29], [30] to determine to what extent the patterns obtained with aggression tests correlated with genetic differentiation between supercolonies. This software infers the number of clusters (*K*) that best fits a data set by maximizing Hardy-Weinberg equilibrium and calculates the assignment probability for each individual under any assumed *K.* We performed ten independent runs for *K* from one to six. All genotyped individuals of a supercolony were included in this analysis.

### Cuticular Hydrocarbon (CHC) Profiles

Cuticular hydrocarbons were extracted from 20 pooled workers from the same nests as those sampled for bioassays (subcolonies) and genetic analysis plus two additional nests for supercolony P2. Single workers did no yield sufficient amounts of substances to allow analysis of the CHC profiles. The individuals were frozen at −20°C for 30 min prior to 8 min extraction in hexane. Extracts were reduced under a gentle stream of nitrogen to 10 to 15 µl and immediately used for analysis or stored at −20°C. 1 µl of the extract was analysed by gas chromatography-mass spectrometry (GC-MS) using a Hewlett-Packard HP 6890 gas chromatograph (GC, equipped with a J & W DB-5 fused silica capillary column: 30 m×0.25 mm ID; film thickness: 0.25 µm) coupled with a Hewlett-Packard HP 5973 mass selective detector (Hewlett-Packard, Waldbronn, Germany). We used a temperature program starting from 60°C with an increase of 5°C/min until a final temperature of 300°C, which was kept for 10 min. A split/splitless injector was set to splitless mode for 60 sec at a temperature of 250°C. Helium was used as carrier gas with a constant flow of 1 ml/min. Electron ionization mass spectra (EI-MS) were recorded at 70 eV with a source temperature of 230°C.

Only hydrocarbons, which were identified by their typical mass spectra, were included in our analysis. Furthermore, all molecules smaller than C19-bodies were discarded, as they only occurred in traces and were absent in most ant CHC profiles [31]. We compared differences in cuticular hydrocarbon profiles between supercolonies by performing permutation tests (adonis, R-package vegan 1.15, 10000 runs) based on relative peak areas (proportion of each peak's integrated peak area to total integrated peak area). To visualize differences in CHC profiles, we performed non-metric multidimensional scaling (NMDS) based on Bray-Curtis dissimilarities (*d*_ij_) of relative peak areas of CHC profiles.

### Correlations between behaviour, genetic and chemical properties of workers from different supercolonies

We constructed a matrix of the proportion of alleles that were not shared by supercolony pairs and, likewise, a matrix of the proportion of qualitatively differing cuticular hydrocarbon substances between supercolony pairs. We then tested for correlation between these two matrices and the matrices of pairwise aggression (*AI*, *MI*), relatedness (*R*) and Bray-Curtis-distances of CHC profiles (*d_ij_*) by performing mantel tests (10000 permutations) between matrix pairs. If not stated otherwise, all statistical analyses were performed with *Statistica* 7.1 (StatSoft, 2005) or R 2.9.2 [32].

## Results

### Behavioural assays

**Figure 1:**
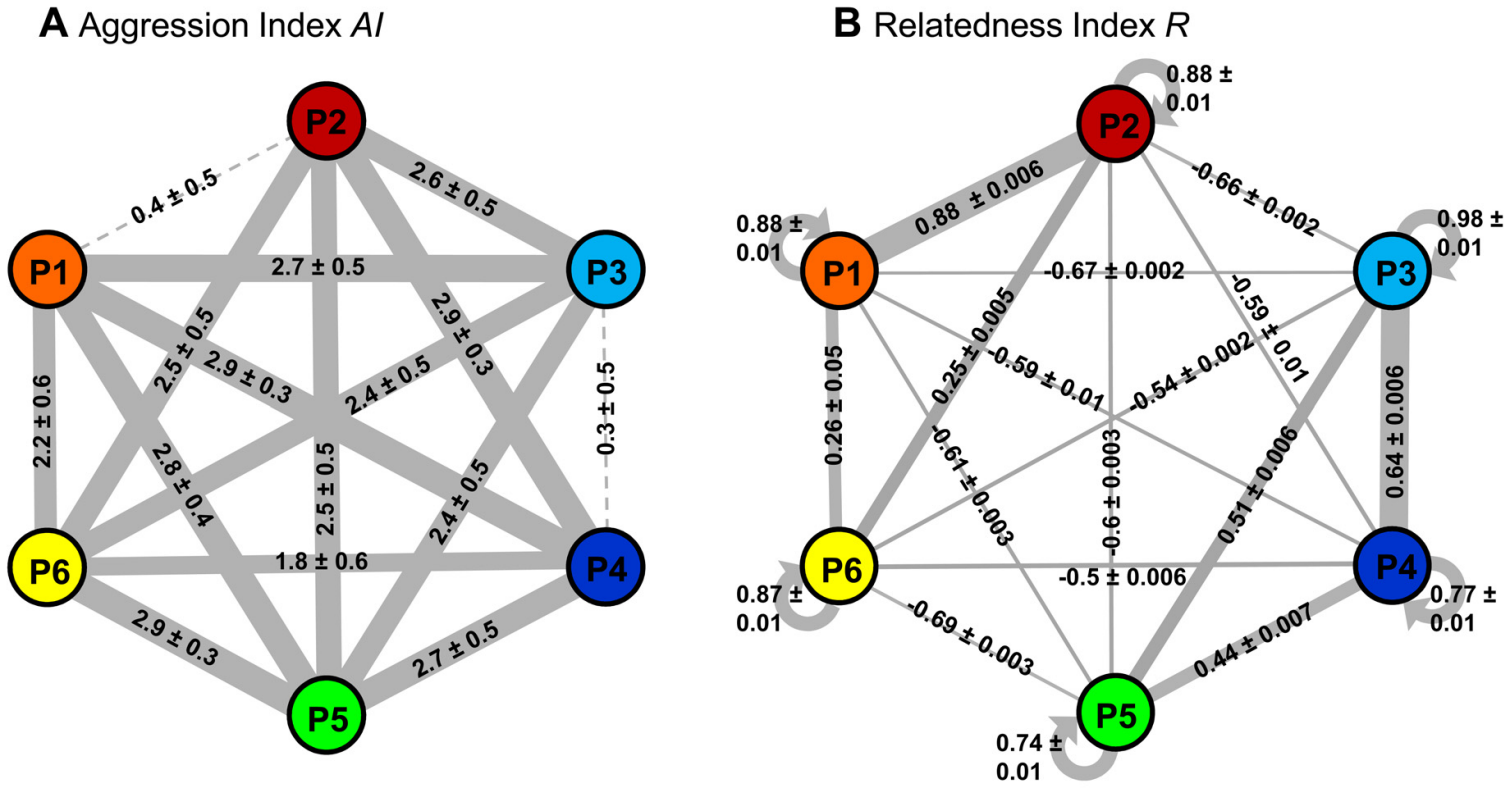
A. Aggression and B. Relatedness between six laboratory colonies collected from six spatially separated *Anoplolepis gracilipes* supercolonies. Aggression was measured as *AI* (mean ± SD, range: 1–4) and relatedness was calculated as *R* (mean ± SE, range: −1–1). The line widths are equivalent to the degree of aggression and relatedness, respectively, and dotted lines represent absent aggression. Colour codes correspond to colony affiliation and to the results of a Bayesian clustering algorithm under the assumption of *K* = 4 genetic clusters (Fig. 2).

Both types of behavioural assays (*AI*, *MI*) yielded consistent results (R = 0.65, p = 0.002, Mantel test). Aggression differed among supercolonies: While workers from P1-P2 and P3-P4 tolerated each other, workers from the remaining pairwise bioassays were aggressive towards each other (Kruskal-Wallis ANOVA, *AI*: H = 177.5, p<0.0001, Fig. 1A; *MI*: H = 144.4, p<0.0001, Figure S2). Assuming that P1-P2 and P3-P4 belonged to the same supercolony, despite being separated up to ca. 1.5 km (P3-P4), we performed the statistical analysis again without colony pairs P1-P2 and P3-P4. Still, aggression differed among the remaining pairs (*AI*: H = 36.1, p = 0.0003, Fig. 1A; *MI*: H = 60.4, p<0.0001, Figure S2). Furthermore, we observed high levels of aggression of workers towards allocolonial sexuals (virgin queens/males) in at least 50% of all replicates in each pairwise supercolony combination (Table S1).

### Relatedness and population structure

**Figure 2:**
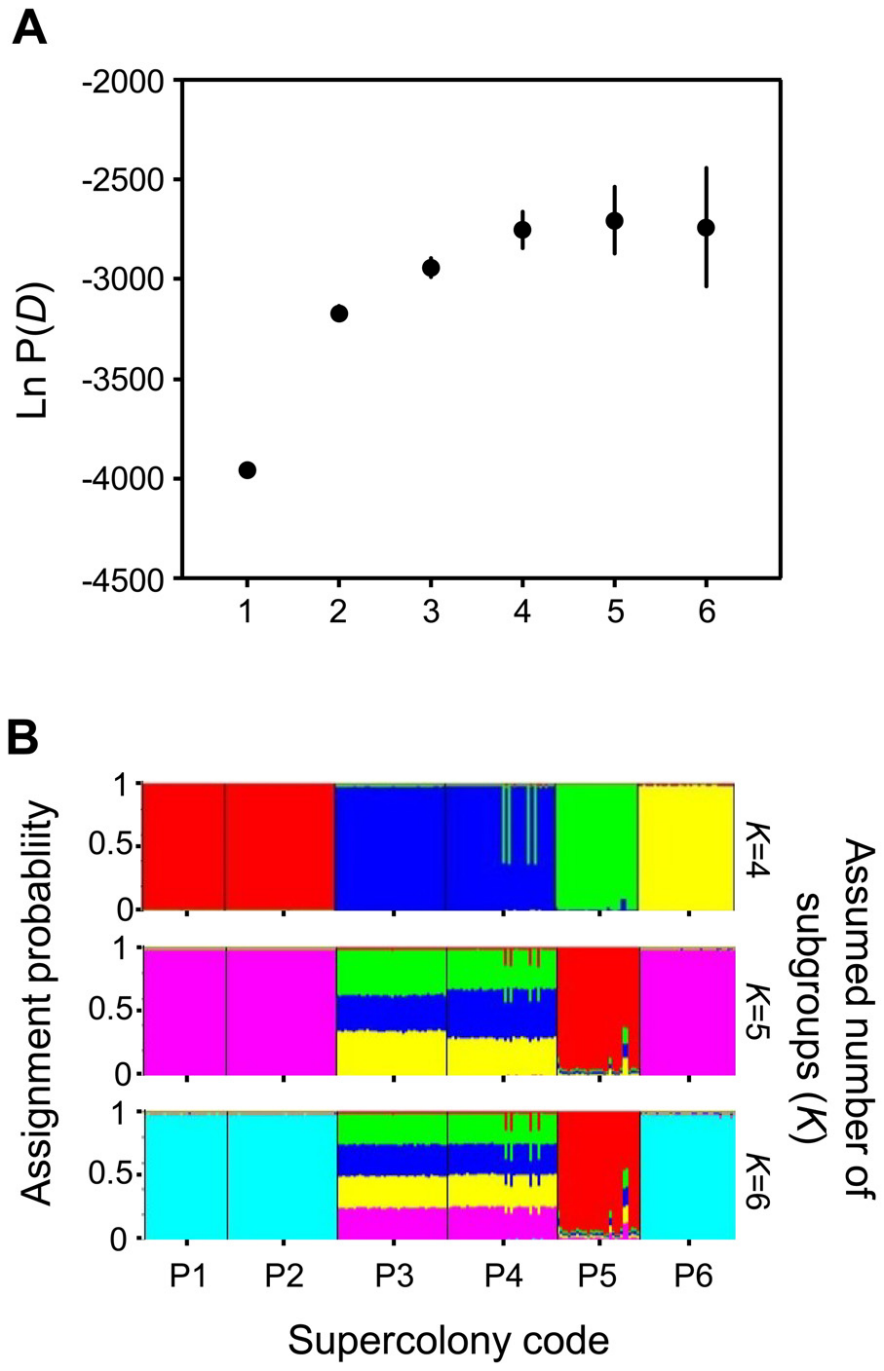
Estimated number of genetic clusters (A) and assignment to four, five and six clusters (B) in a population of six *A. gracilipes* supercolonies. **A.** Average of the logarithm of the likelihood (Ln P(*D*)) of the data to be assigned to *K* genetic clusters as calculated with STRUCTURE 2.2. **B.** Assignment probability of n = 220 *Anoplolepis gracilipes* workers from colonies P1 - P6 to *K* = 4, *K* = 5 and *K* = 6 genetic clusters. Identical colours indicate that subgroups belong to the same genetic cluster. At *K* = 5 and *K* = 6, individuals from colonies P3 and P4 can not be assigned to a specific cluster. At *K* = 4, individuals from colonies P1 and P2 are assigned to the red cluster, individuals from colonies P3 and P4 are assigned to the blue cluster and individuals from colonies P5 and P6 are each assigned an individual cluster.

We found 29 alleles in the entire population (215 individuals from six spatially separated colonies genotyped at six microsatellite loci). The number of alleles per locus ranged from three to 11, while the number of alleles per locus per supercolony ranged between two and four (Table S2). Laboratory subcolonies contained the same alleles as the other genotyped nests from their respective supercolony. Mutually aggressive subcolony pairs shared less than half of their alleles (33.1±17.5%, mean ± SD), while mutually tolerant subcolonies P1-P2 and P3-P4 shared either all alleles (P1-P2) or 85.7% (P3-P4, 16 of 18 alleles in common) of the pairwise allele pool (Table S3). Workers within subcolonies were closely related (*R* = 0.85±0.09, mean ± SE, range: 0.74 — 0.98, Fig. 1B), while relatedness between reciprocally aggressive subcolony pairs was low (*R* = −0.31±0.47, mean ± SE, range: −0.69–0.51). The mean relatedness between the two tolerant supercolony pairs P1-P3 and P3-P4 was as high as intracolonial relatedness (Fig. 1B). Accordingly, the genetic diversity in the population sample originated from differences between supercolonies (AMOVA, F_SCOLONY-TOTAL_  = 0.247, Table S4) rather than differences between individuals in nests (F_IND-NEST_  = −0.925) or nests within supercolonies (F_NEST-SCOLONY_  = −0.002). Without *a priori* information on the origin of the sampled individuals, the Bayesian clustering algorithm implemented in STRUCTURE 2.2 revealed the highest likelihood of the data (Ln P(*D*)) between *K* = 4 and *K* = 6, with *K* = 4 showing the smallest standard deviation (Fig. 2A). When using *a priori* information on assumed numbers of *K*, the assignment of all genotyped individuals to *K* = 4 genetic clusters was clearest, especially for individuals from colonies P3 and P4 (Fig. 2B).

### Cuticular Hydrocarbon (CHC) Profiles

**Figure 3:**
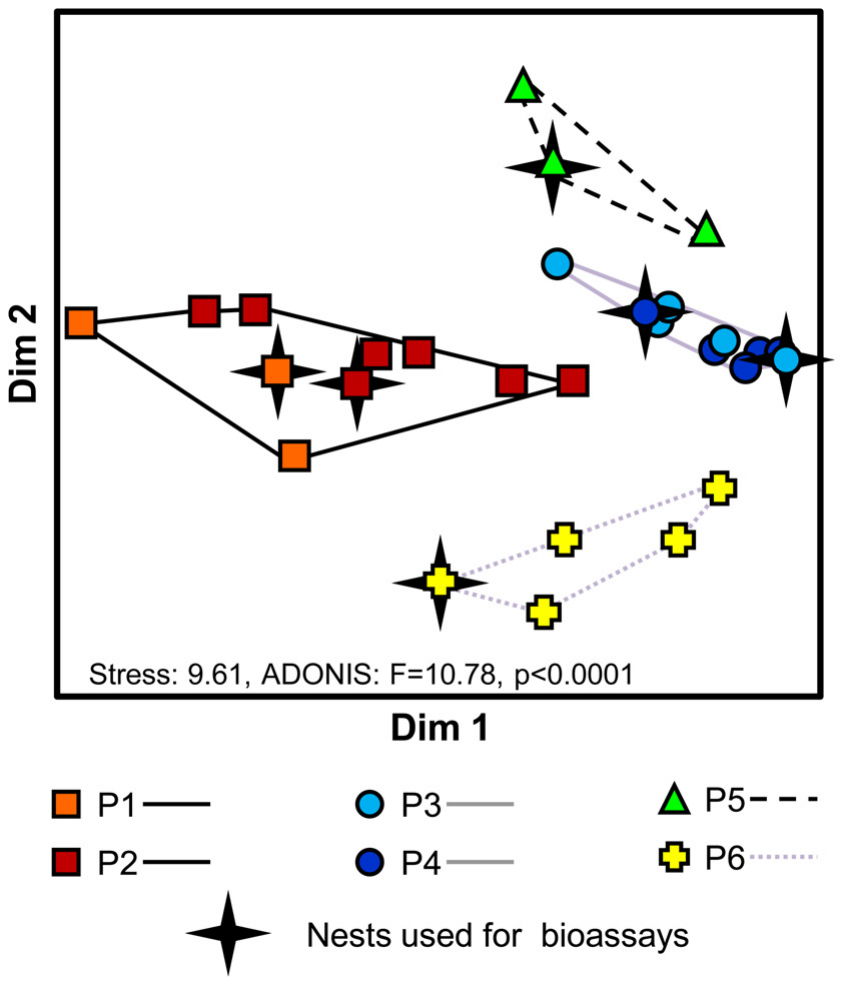
NMDS plot of Bray-Curtis dissimilarities of CHC profiles between six *A. gracilipes* colonies. Three to seven nest sites were included in the analysis. All symbols are coloured according to colony affiliation and the assignment to common genetic clusters (Fig. 2). Colony profiles differ between the four clusters (ADONIS, F = 10.78, p = 0.0001), but not between colonies P1-P2 (F = 2.84, p = 0.11) and P3-P4 (F = 1.80, p = 0.20).

In total, we scored 154 peaks from GC-MS chromatograms of 26 *A. gracilipes* nest sites in Poring Hot Springs. The chemical profiles of pooled workers of reciprocally tolerant subcolonies were much more similar (Bray-Curtis distances d_P1-P2_  = 0.12 and *d*_P3-P4_  = 0.14, respectively) than those of mutually aggressive subcolonies (*d*_ij_ = 0.32±0.06, mean ± SD, range: 0.22–0.42, Figure S3). Likewise, the cuticular hydrocarbon profiles from additional nests of the six *Anoplolepis gracilipes* supercolonies in the field clustered according to supercolony affiliation (ADONIS, F = 10.78, p<0.0001, Fig. 3) and were highly similar between the nests of supercolonies of which subcolonies were mutually tolerant (ADONIS, P1-P2: F = 2.84, p = 0.18; P3-P4: F = 1.80, p = 0.23).

**Figure 4:**
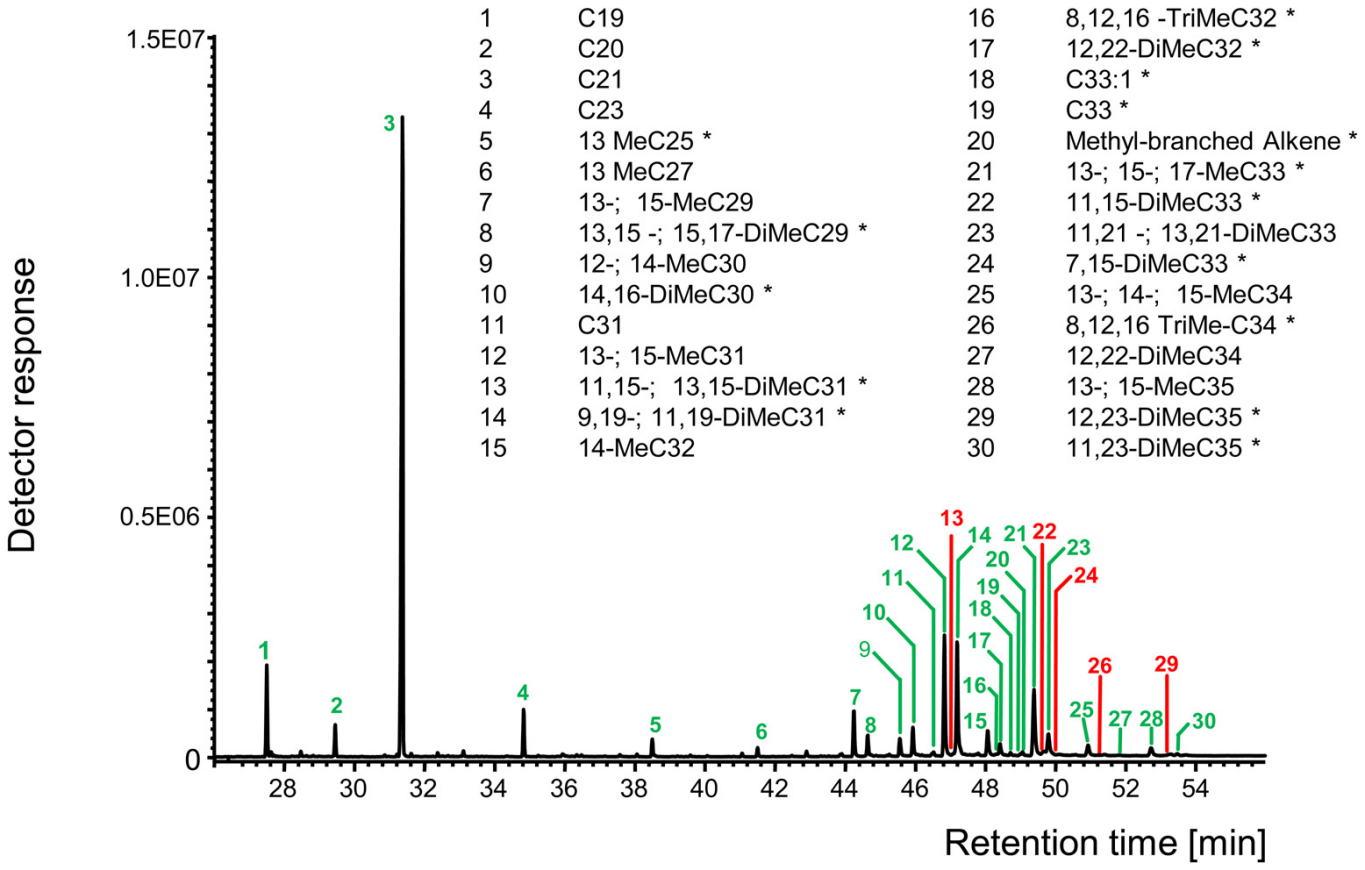
GC-MS chromatogram of a pooled sample of 20 *Anoplolepis gracilipes* workers from supercolony P3. Thirty cuticular substances remained after applying a 1% scoring threshold. Green labels indicate cuticular compounds that are present in this sample, whereas red labels indicate substances that are absent from this sample, but present in cuticular extracts from other supercolonies. Asterisks in the compound list indicate the 16 substances that differ qualitatively between supercolony CHC-profiles.

We then limited the array of analyzed peaks to only those, which constituted at least 1% of relative peak area in at least one sample, thus reducing the array of cuticular compounds from 154 (all substances) to 30. Nevertheless, ADONIS results and NMDS arrangements remained largely the same (Figure S4). The remaining 30 substances comprised a mix of unbranched and methyl-branched alkanes and alkenes, 16 of which differed qualitatively between supercolonies (i.e. compounds that were present in the CHC profiles of one or several supercolonies while being absent in others, Fig. 4). Qualitative differences between CHC profiles involved all identified compound-classes, but were overrepresented in dimethyl-branched alkanes (Fig. 4). Qualitatively differing compounds constituted between 1.01% and 8.21% per compound of the respective CHC profiles (average: 2.59%±0.5%, mean ± SE). Moreover mutually aggressive colonies shared only 73.8% of the compounds in their CHC profiles on average, while mutually tolerant colonies (P1-P2, P3-P4) shared 95.8% of the compounds (presence/absence of compounds, Table S5).

### Correlations between behaviour, genetic and chemical properties of workers from different supercolonies

The behavioural patterns among subcolonies were in perfect agreement with intercolonial relatedness and Bray-Curtis similarities of CHC profiles. Intercolonial aggression (in both *AI* and *MI*) was negatively correlated to relatedness (*AI∼R*: r = −0.68, p = 0.023; *MI*∼*R*: r = −0.84, p = 0.002, Mantel test, Table 1) and positively correlated to Bray-Curtis distances of the CHC profiles (*AI∼d*_ij_: r = 0.72, p = 0.015; *MI*∼*R*: r = 0.62, p = 0.023). Thus, high aggression occurred at low relatedness and high CHC profile dissimilarity, while low aggression matched high relatedness and low dissimilarity of CHC profiles. Accordingly, relatedness and CHC profile dissimilarity were negatively correlated (r = −0.77, p = 0.007). Moreover, the proportion of alleles that differed between supercolonies pairs was positively correlated to the proportion of CHC compounds that differed between supercolony profiles (r = 0.26, p = 0.044).

**Table 1:** Correlation coefficients between aggression, relatedness and chemical and genetic differentiation between six *Anoplolepis gracilipes* supercolonies.

	*AI*	*MI*	*R*	*d_ij_* CHC	% Alleles
***MI***	0.65**				
***R***	−0.68*	−0.84**			
***d_ij_*** ** CHC**	0.72*	0.62*	−0.77**		
**% Alleles**	0.85**	0.45+	−0.36+	0.50+	
**% CHC**'**s**	0.38*	0.05	0.09	0.25	0.26*

Abbreviations represent aggression (*AI*), relatedness (*R*), Bray-Curtis distances of cuticular hydrocarbon profiles (d_ij_ CHC), percentage of distinct alleles (% Alleles) and percentage of distinct CHC compounds (% CHC) between six *Anoplolepis gracilipes* supercolonies. Asterisks indicate p-levels:

+p<0.1,

*p<0.05,

**p<0.01.

## Discussion

The present study revealed a significant positive correlation of aggression with differentiation of the cuticular hydrocarbon profile as well as genetic differentiation among sympatric supercolonies of the Yellow Crazy Ant *Anoplolepis gracilipes* in a study site in NE-Borneo. Intercolonial aggression varied among supercolony pairs, ranging from tolerance to mortal encounters in the bioassays. Mutually aggressive supercolonies were distantly related and showed highly differentiated CHC profiles, while two non-aggressive though spatially separated supercolonies were found to have highly similar CHC profiles and relatedness values that were comparable to those found within supercolonies. Similar patterns have been observed in the Argentine Ant *Linepithema humile*, where mutually tolerant supercolonies from different locations (even from different continents) can be highly similar in genetic and chemical terms, and are thus thought to have been introduced from the same source population [33], [34], [35]. Likewise, supercolonies that are highly differentiated in genetic and chemical terms (even if they are close to each other geographically) are thought to have been introduced from different source populations [36], [37]. Hence, the varying degrees of behaviour as well as genetic and chemical differentiation that we observed in *A. gracilipes* in this study may potentially be explained by the same mechanisms, i.e. supercolony pairs P1-P2 and P3-P4 may have been introduced from the same source population whereas supercolonies P5 and P6 may have been introduced from yet another population, respectively. Alternatively, two tolerant supercolonies may belong to the same supercolony separated by uninhabitable terrain (very likely for the pair P1-P2 which was separated by only 150 m) or one supercolony in a tolerant supercolony pair may have originated from the other through via human-mediated jump-dispersal within the study site (possibly true for P3-P4).

Cuticular hydrocarbon (CHC) profiles have repeatedly been shown to enable ants to discriminate between nestmates and non-nestmates [12], [13], [15]. Typically, differing CHC profiles lead to aggression while similar CHC profiles do not. Aggressive responses towards individuals bearing allocolonial CHC profiles may be evoked according to a threshold rule (e.g. *Cataglyphis iberica*) or may be gradual (e.g. *Myrmica rubra*) (reviewed in [38]). The positive correlation between CHC profile dissimilarity and aggression that we observed between individuals from different supercolonies in *A. gracilipes* may suggest the existence of a graded response mechanism, which has also been found in other invasive ant species such as *Lasius neglectus* [5], *Wasmannia auropunctata* [39] and *Linepithema humile* [14], [40], [41].

Supercolonies of *A. gracilipes* showed both quantitative and qualitative differences in their CHC profiles across various types of hydrocarbons (Fig. 4, Table S4). This is remarkable since in most ant species, CHC profiles tend to differ quantitatively between conspecific colonies and qualitatively between species [15], [42], [43]. For instance, 14 out of 41 epicuticular compounds differed qualitatively between the CHC profiles of six *Formica* species (*Formica* sensu stricto) in a study by Martin *et al*. [44], if the same threshold was applied as in our study. In comparison, 16 out of 30 substances differed qualitatively between the profiles of six different supercolonies of *A. gracilipes* in our study. On occasion, high degrees of qualitative differences between CHC profiles of colonies from non-invasive ant species have been reported, but analyses either include all substances (including trace substances, e.g. [43]) or qualitative differences are only detected in one substance class, e.g. homologous series of (Z)-9-alkenes or positional isomers of dimethyl-branched C25-bodies [45]. In invasive ant species, qualitative differences between CHC profiles of different supercolonies have repeatedly been found, e.g. between invasive and native populations of *Linepithema humile* [35] and *Wasmannia auropunctata* [39]. CHC profiles were found to be less complex in introduced areas, with substances having been lost from the profiles in comparison to colonies in the native range [39]. However, supercolonies of *Linepithema humile* also differ in their CHC profiles in the invasive range [41].

Here, we found unusually high degrees of qualitative differences between CHC profiles of different supercolonies of *A. gracilipes*. We identified 30 major CHC compounds that accounted for at least 1% of the entire CHC profile and found qualitative differences in compounds that accounted for up to 8.21% of the profiles of different *A. gracilipes* supercolonies. The qualitatively differing substances comprised different compound classes such as alkanes, alkenes, methyl-branched alkenes as well as mono-, di- and trimethyl-branched alkanes of varying chain length (Fig. 4), as would be expected if supercolonies diverge in a drift-like process of neutral evolution.

In our study, genetic distances corresponded to chemical distances between colonies, as has been observed in several other studies on various social insect species [5], [46], [47], [48]. The correlation between differentiation in CHC profile and differentiation in microsatellite loci in *A. gracilipes* suggests that cuticular hydrocarbons are to a large extent genetically determined in this species as is in many other ant species [17], [18], [19], [20], [21], albeit environmental cues may also be involved [49], [50].

Helanterä *et al.* suggested that unicoloniality (the ability of ant species to form populations consisting of one or more supercolonies [8], [22]), might be an evolutionary dead-end [22], since selfish behaviour of distantly related nestmates should lead to supercolony instability as predicted by kin-selection theory. This view is supported by the scattered distribution of unicoloniality in the ant phylogeny as well as the fact that no unicolonial ant species has a sister species that is also unicolonial [22]. In contrast to other supercolonial ant species such as *L. humile* and *S. invicta*, however, workers in *A. gracilipes* supercolonies are closely related [22], [23], and thus selfish behaviour is less likely to evolve.

Despite this potential evolutionary dead-end, the limited or even absent gene flow between supercolonies of unicolonial ant species [2], [8], [9], [10] may be conducive to speciation [22]. As speciation occurs on timescales beyond reach for current research, indirect approaches are the only way to find clues regarding that question, e.g. by measuring how much supercolonies have diverged in neutral or selected traits [22]. As we have shown, *A. gracilipes* supercolonies are indeed differentiated in neutral traits, i.e. alleles of microsatellite loci and several cuticular compounds. Regardless of the origin of this initial differentiation that we see today, one should expect both the genetic and chemical differentiation to increase in the prolonged absence of gene flow between supercolonies. Although we cannot infer absence of gene flow directly, the sociogenetic structure of the supercolonies in NE-Borneo resembles that of the supercolonies found on Christmas Island, where gene flow among the two supercolonies can be excluded based on mitochondrial and microsatellite markers [10]. In addition, behavioural characteristics of *A. gracilipes*, such as intranidal mating soon after eclosion of female reproductives (pers. obs.) and aggression towards allocolonial sexuals by workers (Table S1), suggest that mating is non-random, resulting in strongly reduced or absent gene flow between supercolonies. Similar patterns (intranidal mating, limited or absent intercolonial gene flow) have been reported for the likewise invasive ant *Linepithema humile* both in its native [8], [48] and invasive range [2], [9], [51].

The initial differences that would be necessary in order for subsequent differentiation between neighbouring supercolonies to occur are likely to accumulate in allopatry, e.g. if supercolonies stem from different source populations or if supercolonies are divided into two or more fragments through human-mediated jump dispersal. In the latter scenario, lack of contact zones (and thus mating) between the resulting allopatric fragments is likely to lead to accumulation of colony (or fragment-) specific mutations and CHC compounds. If both fragments come into secondary contact again, genetic and chemical differentiation may have advanced enough to result in mutual aggression and lack of gene flow. This pattern was very recently described for *Linepithema humile* supercolonies from Corsica and the European mainland, where the introduction of one or several *L. humile* propagules from the mainland to Corsica likely entailed an interruption of gene flow between Corsican and European supercolonies, resulting in the pronounced chemical and behavioural differentiation observable today [52]. This scenario may also explain parts of our data, as supercolonies P3 and P4 are likely to be established fragments of the same supercolony which have already accumulated supercolony-specific alleles and cuticular compounds despite still tolerating each other (Tables S3 and S5). Over time, the spatial separation (allopatry) of the two supercolonies P3 and P4 should facilitate increasing genetic and chemical differentiation, which in turn should lead to mutual aggression as soon as CHC profiles are sufficiently different. Furthermore, genetic and chemical differentiation should also entail behavioural suppression of intercolonial gene flow through worker aggression against allocolonial sexuals even if they came into contact again (e.g. as a result of range expansion).

Thus, we suggest that the combination of exclusive intranidal mating and budding on the one hand and positive feedback between genetic, chemical and behavioural traits on the other hand may drive supercolonies towards ever increasing differentiation, possibly even involving reproductive isolation and thus, speciation. In contrast to the majority of ant species, *A. gracilipes* supercolonies are characterized by a combination of traits (polygyny, intranidal mating, and lack of active dispersal other than budding) that ensure the continuous production of generation upon generation of reproductives that all stay within the supercolony. Coupled with an unusual reproductive system that may potentially avoid the negative effects of inbreeding depression [23], the extreme polygyny and polydomy of *A. gracilipes* immensely reduces the risk of a breakdown of the entire supercolony, resulting in virtual immortality of the supercolony superorganism. Furthermore, in eusocial species with strict intranidal mating, a colony-specific signal should be sufficient for recognition between the sexes and thus, selection towards a species-specific signal that allows recognition between sexes from different supercolonies should be relaxed [53]. In concert with the virtual immortality of *A. gracilipes* supercolonies, relaxed selection towards a species-specific signal may even lead to a complete breakdown of intraspecific recognition between sexuals from different colonies, potentially leading to prezygotic, reproductive isolation. This, in turn, implies that different *A. gracilipes* supercolonies would no longer belong to the same species according to the biological concept of species, which states that species are groups of interbreeding natural populations that are reproductively isolated from other such groups [54]. Currently it is unclear whether different *A. gracilipes* supercolonies should be perceived as belonging to the same species, as apparent lack of random mating between sexuals from different supercolonies, intranidal mating and aggression of workers towards allocolonial sexuals may already pose a sufficient barrier preventing intercolonial gene flow. Thus, we argue that intercolonial differentiation may potentially result in reproductive barriers between colonies, ultimately leading to independent units on different evolutionary trajectories, and thus possibly speciation between neighbouring *A. gracilipes* supercolonies.

## Supporting Information

Figure S1 Location of six *Anoplolepis gracilipes* supercolonies in Sabah, Malaysia.
(0.37 MB PDF)

Figure S2 Aggression between six laboratory colonies collected from six spatially separated *Anoplolepis gracilipes* supercolonies.
(0.37 MB PDF)

Figure S3 Bray-Curtis dissimilarities of relative peak areas of cuticular hydrocarbon profiles between six *Anoplolepis gracilipes* supercolonies.
(0.26 MB PDF)

Figure S4 NMDS plots of Bray-Curtis dissimilarities of CHC profiles of six *Anoplolepis gracilipes* supercolonies.
(0.38 MB PDF)

Text S1 Materials and Methods: Mortality Index *MI*.
(0.18 MB PDF)

Table S1 Aggression of workers towards allocolonial workers, males and queens.
(0.14 MB PDF)

Table S2 Alleles of six microsatellite loci in six *Anoplolepis gracilipes* supercolonies.
(0.17 MB PDF)

Table S3 Percentage of alleles differing between *Anoplolepis gracilipes* supercolonies in relation to the pairwise allele pool.
(0.10 MB PDF)

Table S4 Multi-level genetic analysis of molecular variance (AMOVA) of six *Anoplolepis gracilipes* supercolonies.
(0.13 MB PDF)

Table S5 Percentage of CHC differing between six *Anoplolepis gracilipes* supercolonies in relation to the pairwise compound pool.
(0.12 MB PDF)
