# Supplementary material for: Societies Drifting Apart? Behavioural, Genetic and Chemical Differentiation between Supercolonies in the Yellow Crazy Ant Anoplolepis gracilipes
Source: PLoS One. 2010 Oct 22;5(10):e13581. doi: 10.1371/journal.pone.0013581 (PMC2962633; doi:10.1371/journal.pone.0013581)
Supplement: Figure S1 — Location of six Anoplolepis gracilipes supercolonies in Sabah, Malaysia. (0.37 MB PDF) [file pone.0013581.s001.pdf]

Societies Drifting Apart? Behavioural, Genetic and Chemical Differentiation Between Supercolonies in the Yellow Crazy Ant *Anoplolepis gracilipes*

Jochen Drescher, Nico Blüthgen, Thomas Schmitt, Jana Bühler, Heike Feldhaar

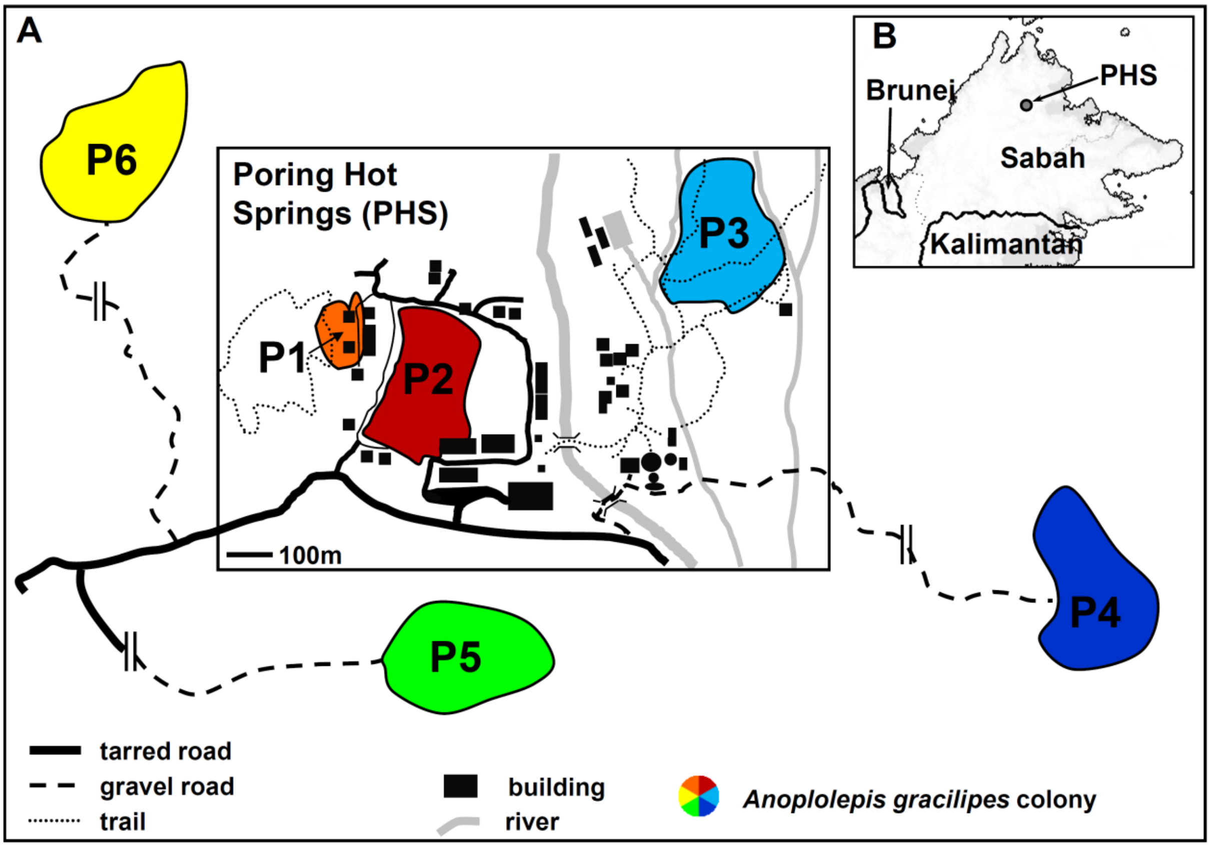

Fig. S1 Location of six *Anoplolepis gracilipes* supercolonies in Sabah, Malaysia.
A. Location and estimated size of the six *Anoplolepis gracilipes* colonies in the study area (Poring Hot Springs, PHS). The area outside of the box is not drawn to scale. B. Location of Poring Hot Springs in North East Borneo. Colour codes correspond to colony affiliation and to the results of a Bayesian clustering algorithm under the assumption of K=4 genetic clusters (Fig. 2, main document).
