# Supplementary material for: Societies Drifting Apart? Behavioural, Genetic and Chemical Differentiation between Supercolonies in the Yellow Crazy Ant Anoplolepis gracilipes
Source: PLoS One. 2010 Oct 22;5(10):e13581. doi: 10.1371/journal.pone.0013581 (PMC2962633; doi:10.1371/journal.pone.0013581)
Supplement: Figure S3 — Bray-Curtis dissimilarities of relative peak areas of cuticular hydrocarbon profiles between six Anoplolepis gracilipes supercolonies. (0.26 MB PDF) [file pone.0013581.s003.pdf]

Societies Drifting Apart? Behavioural, Genetic and Chemical Differentiation Between Supercolonies in the Yellow Crazy Ant *Anoplolepis gracilipes*

Jochen Drescher, Nico Blüthgen, Thomas Schmitt, Jana Bühler, Heike Feldhaar

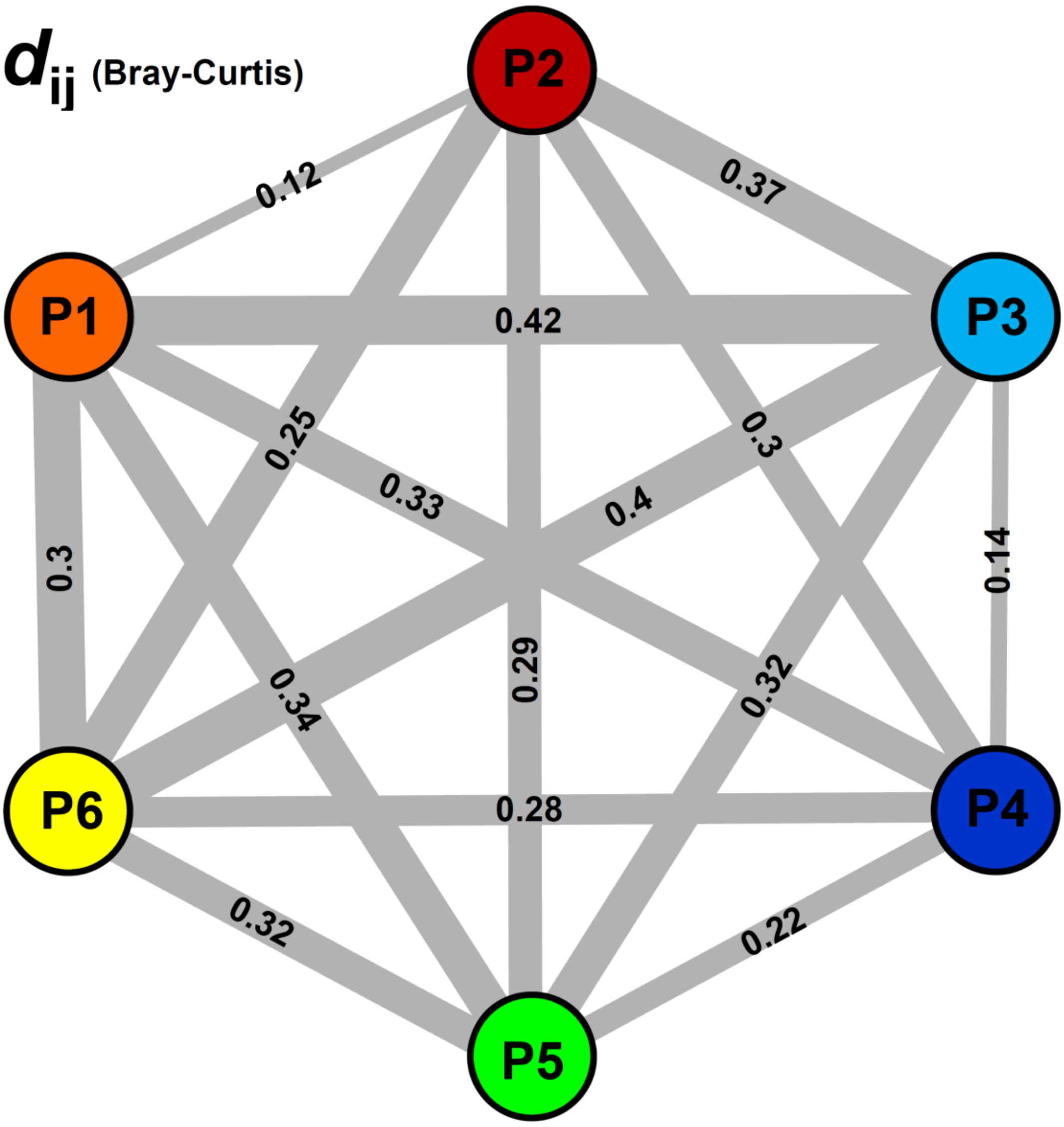

Figure S3 Bray-Curtis dissimilarities of relative peak areas of cuticular hydrocarbon profiles between six *Anoplolepis gracilipes* supercolonies. Colour codes correspond to colony affiliation and to the results of a Bayesian clustering algorithm under the assumption of *K*=4 genetic clusters (Fig. 2, main document).
