# Supplementary material for: Societies Drifting Apart? Behavioural, Genetic and Chemical Differentiation between Supercolonies in the Yellow Crazy Ant Anoplolepis gracilipes
Source: PLoS One. 2010 Oct 22;5(10):e13581. doi: 10.1371/journal.pone.0013581 (PMC2962633; doi:10.1371/journal.pone.0013581)
Supplement: Figure S4 — NMDS plots of Bray-Curtis dissimilarities of CHC profiles of six Anoplolepis gracilipes supercolonies. (0.38 MB PDF) [file pone.0013581.s004.pdf]

Societies Drifting Apart? Behavioural, Genetic and Chemical Differentiation Between Supercolonies in the Yellow Crazy Ant *Anoplolepis gracilipes*

Jochen Drescher, Nico Blüthgen, Thomas Schmitt, Jana Bühler, Heike Feldhaar

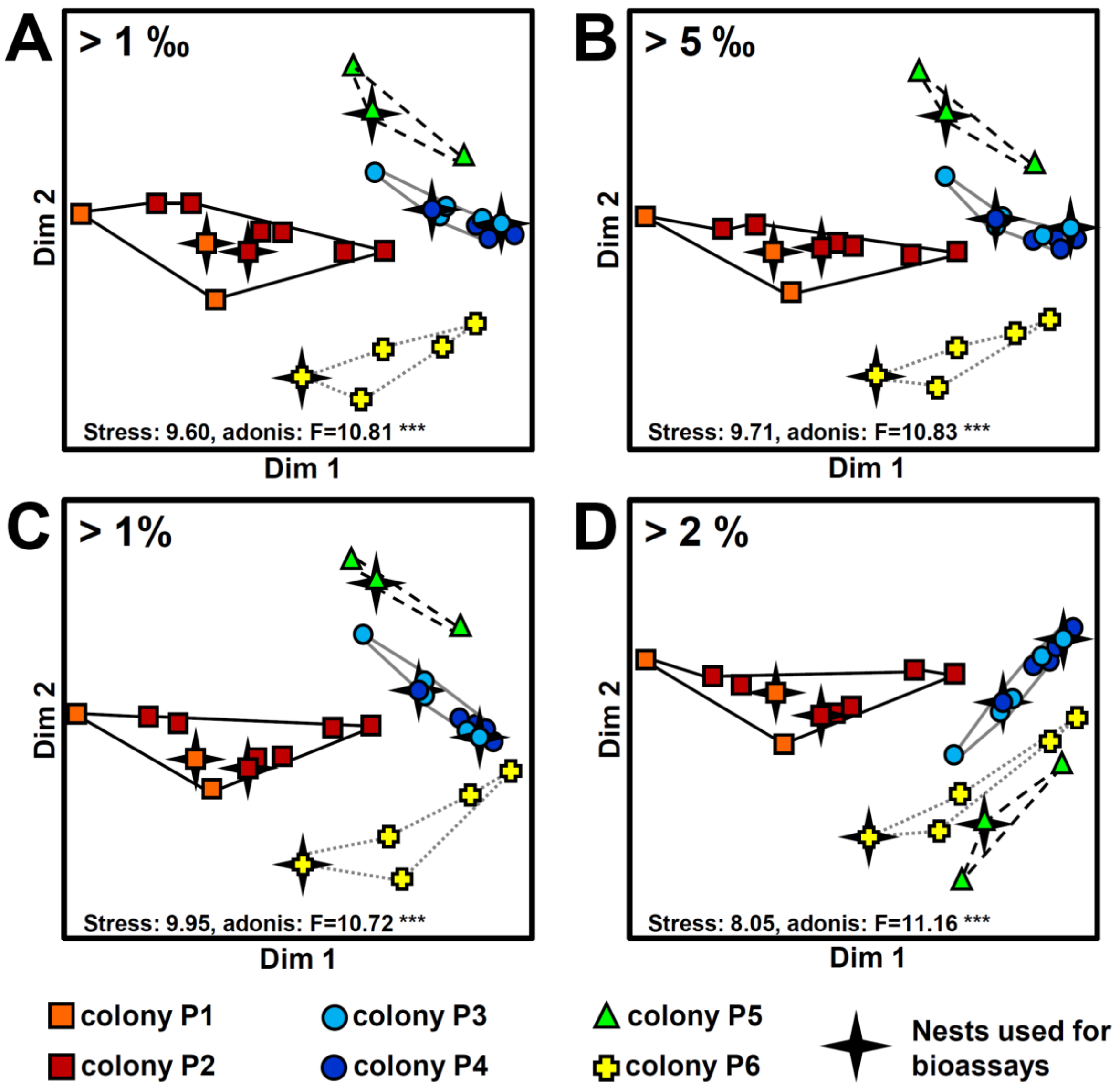

Figure S4 NMDS plots of Bray-Curtis dissimilarities of CHC profiles of six *A. gracilipes* supercolonies. We calculated Bray-Curtis dissimilarities of the CHC profiles using four different scoring thresholds (A-D).A. Relative peak areas (RPA) were larger than 1‰ in at least one sample. B. RPA's larger than 5‰ in at least one sample. C. RPA's larger than 1% in at least one sample. D. RPA's larger than 2% in at least one sample. Colour codes correspond to colony affiliation and to the results of a Bayesian clustering algorithm under the assumption of *K*=4 genetic clusters (Fig. 2, main document). While adonis results and stress values of the NMDS plots remained similar regardless of the predefined thresholds, the arrangements of NMDS plots were similar to the original arrangement at thresholds >1‰, >5‰ and >1% (A-C), but not at >2% (D).
