# Supplementary material for: Societies Drifting Apart? Behavioural, Genetic and Chemical Differentiation between Supercolonies in the Yellow Crazy Ant Anoplolepis gracilipes
Source: PLoS One. 2010 Oct 22;5(10):e13581. doi: 10.1371/journal.pone.0013581 (PMC2962633; doi:10.1371/journal.pone.0013581)
Supplement: Text S1 — Materials and Methods: Mortality Index MI. (0.18 MB PDF) [file pone.0013581.s005.pdf]

Societies Drifting Apart? Behavioural, Genetic and Chemical Differentiation Between Supercolonies in the Yellow Crazy Ant *Anoplolepis gracilipes*

Jochen Drescher, Nico Blüthgen, Thomas Schmitt, Jana Bühler, Heike Feldhaar

Text S1 Materials and Methods: Mortality Index *MI*

We measured aggression of workers towards allocolonial sexuals, i.e. virgin alate queens and males. As not enough virgin queens and males could be collected from the supercolonies in Poring Hot Springs, these tests were performed within and between three supercolonies from the Sepilok Forest Reserve (supercolonies S7, S8 and S10, 5°52’ N, 117°57’ E) and one from the Lower Kinabatangan Nature Reserve (supercolony K1, 5°29’ N, 118°16’ E), Sabah, Malaysia. Aggression towards allocolonial sexuals in a combination of supercolonies was measured by placing five workers from one supercolony and either one alate queen or one male from another supercolony in an arena (see main document) and vice versa. We then scored presence/absence of aggression (biting, spread-eagling of the queen/male, protruding gaster and spraying acid) in 10 trials of 60 min each. Overall aggression towards allocolonial virgin queens/males was obtained by pooling the data from the 20 replicates per reproductive caste per supercolony combination. Aggression between workers of the same supercolonies was measured similarly, only that five individuals of each supercolony were placed in the arena (N=10 replicates per supercolony combination).
