# Supplementary material for: Societies Drifting Apart? Behavioural, Genetic and Chemical Differentiation between Supercolonies in the Yellow Crazy Ant Anoplolepis gracilipes
Source: PLoS One. 2010 Oct 22;5(10):e13581. doi: 10.1371/journal.pone.0013581 (PMC2962633; doi:10.1371/journal.pone.0013581)
Supplement: Table S1 — Aggression of workers towards allocolonial workers, males and queens. (0.14 MB PDF) [file pone.0013581.s006.pdf]

Societies Drifting Apart? Behavioural, Genetic and Chemical Differentiation Between Supercolonies in the Yellow Crazy Ant *Anoplolepis gracilipes*

Jochen Drescher, Nico Blüthgen, Thomas Schmitt, Jana Bühler, Heike Feldhaar

Table S1 Aggression of workers towards allocolonial workers, males and queens. The number of crosses represent the frequency of aggression of workers towards allocolonial queens (red crosses), males (blue crosses) and workers (black crosses) from four different *Anoplolepis gracilipes* supercolonies (supercolonies K1, S6, S7and S10; ++: aggression was observed in more than 50% of all replicates; +++: aggression observed in more than 75% of all replicates).

	**K1**	**S6**	**S7**	**S10**
**K1**	–	+++/+++	++/++	++/++
**S6**	+++	–	++/++	+++/++
**S7**	++	++	–	++/++
**S10**	++	+++	+++	–
