# Supplementary material for: Societies Drifting Apart? Behavioural, Genetic and Chemical Differentiation between Supercolonies in the Yellow Crazy Ant Anoplolepis gracilipes
Source: PLoS One. 2010 Oct 22;5(10):e13581. doi: 10.1371/journal.pone.0013581 (PMC2962633; doi:10.1371/journal.pone.0013581)
Supplement: Table S2 — Alleles of six microsatellite loci in six Anoplolepis gracilipes supercolonies. (0.17 MB PDF) [file pone.0013581.s007.pdf]

Societies Drifting Apart? Behavioural, Genetic and Chemical Differentiation Between Supercolonies in the Yellow Crazy Ant *Anoplolepis gracilipes*

Jochen Drescher, Nico Blüthgen, Thomas Schmitt, Jana Bühler, Heike Feldhaar

Table S2 Alleles of six microsatellite loci in six *Anoplolepis gracilipes* supercolonies. The alleles of six microsatellite loci (Ano1, Ano3, Ano4, Ano6, Ano8, Ano10) are given in rows, the six *Anoplolepis gracilipes* supercolonies (P1-P6) and the entire study area (Total) are given in columns. N^n indicates the number of nests and N^w the number of workers genotyped per supercolony.

	P1	P2	P3	P4	P5	P6	Total
**Ano1**	99<br>103	99<br>103	99<br>103	99<br>103	99<br>103	99<br>105	99<br>103<br>105
**Ano3**	164<br>166	164<br>166	144<br>156	144<br>156	144<br>156	164<br>166<br>168	144<br>156<br>164<br>166<br>168
**Ano4**	159<br>175	157<br>175	157<br>165	157<br>165	157<br>165	159<br>175	157<br>159<br>165<br>175
**Ano6**	116<br>130	116<br>130	116<br>130	116<br>130	116<br>118<br>130	116<br>130	116<br>118<br>130
**Ano8**	214<br>224<br>294	214<br>224<br>294	220<br>274<br>278	212<br>220<br>278	208<br>212<br>260<br>274	220<br>286<br>292	208<br>212<br>214<br>220<br>224<br>260<br>274<br>278<br>286<br>292<br>294
**Ano10**	234<br>242	234<br>242	234<br>238	234<br>238	234<br>238	234<br>242	234<br>238<br>242
**N^n**	3	5	5	5	3	5	26
**N^w**	30	40	40	40	30	40	220
