# Supplementary material for: Societies Drifting Apart? Behavioural, Genetic and Chemical Differentiation between Supercolonies in the Yellow Crazy Ant Anoplolepis gracilipes
Source: PLoS One. 2010 Oct 22;5(10):e13581. doi: 10.1371/journal.pone.0013581 (PMC2962633; doi:10.1371/journal.pone.0013581)
Supplement: Table S3 — Percentage of alleles differing between Anoplolepis gracilipes supercolonies in relation to the pairwise allele pool. (0.10 MB PDF) [file pone.0013581.s008.pdf]

Societies Drifting Apart? Behavioural, Genetic and Chemical Differentiation Between Supercolonies in the Yellow Crazy Ant *Anoplolepis gracilipes*

Jochen Drescher, Nico Blüthgen, Thomas Schmitt, Jana Bühler, Heike Feldhaar

Table S3 Percentage of alleles differing between *Anoplolepis gracilipes* supercolonies in relation to the pairwise allele pool.

	**P1**	**P2**	**P3**	**P4**	**P5**
**P2**	0				
**P3**	76.2	76.2			
**P4**	76.2	76.2	14.3		
**P5**	78.3	78.3	35.3	35.3	
**P6**	50	50	77.3	77.3	84
