# Supplementary material for: Societies Drifting Apart? Behavioural, Genetic and Chemical Differentiation between Supercolonies in the Yellow Crazy Ant Anoplolepis gracilipes
Source: PLoS One. 2010 Oct 22;5(10):e13581. doi: 10.1371/journal.pone.0013581 (PMC2962633; doi:10.1371/journal.pone.0013581)
Supplement: Table S4 — Multi-level genetic analysis of molecular variance (AMOVA) of six Anoplolepis gracilipes supercolonies. (0.13 MB PDF) [file pone.0013581.s009.pdf]

Societies Drifting Apart? Behavioural, Genetic and Chemical Differentiation Between Supercolonies in the Yellow Crazy Ant *Anoplolepis gracilipes*

Jochen Drescher, Nico Blüthgen, Thomas Schmitt, Jana Bühler, Heike Feldhaar

Table S4 Multi-level genetic analysis of molecular variance (AMOVA) of six *Anoplolepis gracilipes* supercolonies. *: $p < 0.05$; **: $p<0.01$; ***: $p < 0.001$. Significance values were obtained by bootstrapping 10000 times over loci.

	$F_{IND\text{-}NEST}$	$F_{NEST\text{-}SCOLONY}$	$F_{SCOLONY\text{-}TOTAL}$
Ano1	-1	0	**0.141*** **
Ano3	-0.923	-0.003	**0.361*** **
Ano4	-1	0	**0.376*** **
Ano6	-0.945	-0.001	**0.009* **
Ano8	-0.712	-0.009	**0.373*** **
Ano10	-1	0	**0.231*** **
**All**	-0.925	-0.002	**0.247*** **
