# Supplementary material for: Societies Drifting Apart? Behavioural, Genetic and Chemical Differentiation between Supercolonies in the Yellow Crazy Ant Anoplolepis gracilipes
Source: PLoS One. 2010 Oct 22;5(10):e13581. doi: 10.1371/journal.pone.0013581 (PMC2962633; doi:10.1371/journal.pone.0013581)
Supplement: Table S5 — Percentage of CHC differing between six Anoplolepis gracilipes supercolonies in relation to the pairwise compound pool. (0.12 MB PDF) [file pone.0013581.s010.pdf]

Societies Drifting Apart? Behavioural, Genetic and Chemical Differentiation Between Supercolonies in the Yellow Crazy Ant *Anoplolepis gracilipes*

Jochen Drescher, Nico Blüthgen, Thomas Schmitt, Jana Bühler, Heike Feldhaar

Table S5 Percentage of CHC's differing between six *Anoplolepis gracilipes* supercolonies in relation to the pairwise compound pool.

	P1	P2	P3	P4	P5
**P2**	4.2				
**P3**	12	8			
**P4**	8.3	12	4.2		
**P5**	37.9	34.5	34.5	37.9	
**P6**	33.3	29.6	29.6	33.3	29.6
